# In Situ Production and Application of Cellulose Nanofibers to Improve Recycled Paper Production

**DOI:** 10.3390/molecules24091800

**Published:** 2019-05-09

**Authors:** Ana Balea, Jose Luis Sanchez-Salvador, M. Concepcion Monte, Noemi Merayo, Carlos Negro, Angeles Blanco

**Affiliations:** 1 Department of Chemical Engineering and Materials, Universidad Complutense de Madrid (UCM), Av. Complutense s/n, 28040 Madrid, Spain; anabalea@ucm.es (A.B.); josanc03@ucm.es (J.L.S.-S.); cmonte@ucm.es (M.C.M.); nmerayoc@ucm.es (N.M.); cnegro@ucm.es (C.N.); 2 Department of Mechanical, Chemical and Industrial Design Engineering, ETSIDI, Universidad Politécnica de Madrid (UPM), Ronda de Valencia 3, 28012 Madrid, Spain

**Keywords:** nanocellulose, cellulose nanofibers, recycled paper, mechanical properties, drainage, retention, circular economy

## Abstract

The recycled paper and board industry needs to improve the quality of their products to meet customer demands. The refining process and strength additives are commonly used to increase mechanical properties. Interfiber bonding can also be improved using cellulose nanofibers (CNF). A circular economy approach in the industrial implementation of CNF can be addressed through the in situ production of CNF using side cellulose streams of the process as raw material, avoiding transportation costs and reducing industrial wastes. Furthermore, CNF fit for use can be produced for specific industrial applications.This study evaluates the feasibility of using two types of recycled fibers, simulating the broke streams of two paper machines producing newsprint and liner for cartonboard, to produce in situ CNF for direct application on the original pulps, old newsprint (ONP), and old corrugated container (OCC), and to reinforce the final products. The CNF were obtained by 2,2,6,6-tetramethyl-1-piperidinyloxy (TEMPO)-mediated oxidation and homogenization at 600 bar. Handsheets were prepared with disintegrated recycled pulp and different amounts of CNF using a conventional three-component retention system. Results show that 3 wt.% of CNF produced with 10 mmol of NaClO per gram of dry pulp improve tensile index of ONP ~30%. For OCC, the same treatment and CNF dose increase tensile index above 60%. In both cases, CNF cause a deterioration of drainage, but this effect is effectively counteracted by optimising the retention system.

## 1. Introduction

Papermaking is an industrial sector characterized by its commitment to develop sustainable production processes [1,2]. In Europe, 52.4% of the papermaking industry’s raw materials come from recovered paper, which corresponds to a paper recycling rate of 72.3% [3]. Nevertheless, the quality levels required in the utilization of secondary fibers are continuously increasing according to the customer demands. Besides, paper consumption has decreased due to the replacement of paper by other supports for the information, causing cost pressures in the paper and board industry. Despite the fact that natural and synthetic strength additives are commonly used in recycled paper, the main source of complaints is still the poor tensile strength. Therefore, other strategies to improve interfiber bonding have been explored, and the use of cellulose nanofibers (CNF) is a promising alternative to increase mechanical properties of recycled products with some additional advantages, such as their renewable nature, biodegradability, high surface area, and high availability.

CNF have gained more attention due to their high strength and stiffness joined to the low weight [4,5]. For these reasons, CNF is promising in multiple sectors such as papermaking [6], composites [7,8], cement [9], packaging [10], electronic devices [11], coatings, biomedicine [12], or automotives [13]. Regarding the papermaking industry, CNF can improve paper quality, and many studies have shown that their addition to the pulp suspension increases the mechanical properties of the recycled paper [6,14,15,16]. The majority of CNF applied in papermaking as strength additive are produced from virgin pulp [17,18,19], but also from pulps from valueless agriculture residues [15,20,21]. Recently, nanocellulosic materials from papermaking streams such as solid waste from a dissolving cellulose pulp mill have been studied. Jonoobi et al. (2012) [22] produced and characterized these nanofibers as a potential biobased nanomaterial for different applications, but they did not study their use as reinforcement agent in the papermaking process. On the other hand, Campano et al. (2016) [23,24] isolated cellulose nanocrystals (CNC) from recycled newspaper and evaluated their effect on the recycled paper enhancement achieving increments of up to 30% in the tensile strength index when 3 wt.% of CNC was added into the recycled pulp; long pulping times and a polyacrylamide-based retention system were used. In situ produced bacterial cellulose (BC) in recycled pulps has also achieved increments in both tensile and tear indexes of 12.2% and 14.2%, respectively, when BC was produced in agitation culture [25]. However, to the best of our knowledge, there are no studies of the use of CNF produced in situ from recycled paper, simulating the broke streams of the recycled papermaking process, and, subsequently, evaluating their effect as strength agent in the same industrial process.

**Figure 1:**
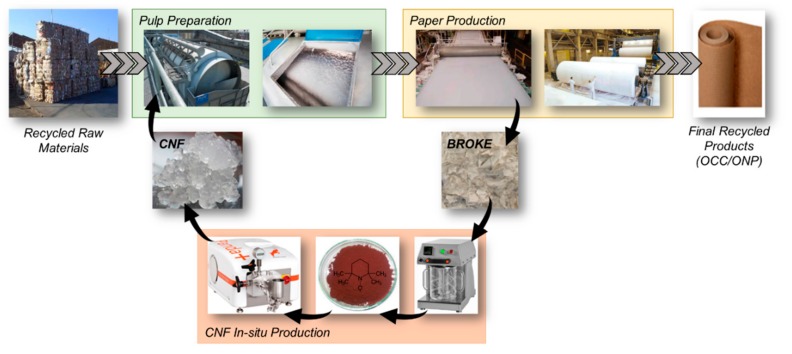
In situ production of cellulose nanofibers (CNF) from recycled papermaking streams to be used as additive in the process.

Large-scale production of CNF is still very limited and produced from virgin pulp. Therefore, the industrial implementation of CNF in the papermaking industry is still a challenge [26]. The main drawbacks to use CNF in large volume applications, such as papermaking, is their cost due to both the high amount of energy required and transportation, the difficulties in producing uniform nanocellulosic particles and the difficulties associated with both dewatering and pumping [6,27]. Some of those drawbacks can be addressed and eventually avoided through the implementation of a circular approach that will lead to an even more sustainable papermaking process. In order to achieve this, one of the key points is the in situ production of CNF (Figure 1) using process and waste cellulose streams, such as fines-rich streams, coming from the filtering of the screw presses or from the white-waters, dry and wet broke from the paper machine, and rejects from the flotation processes. Some of the advantages associated with this approach are the increase in the yield of the process, the avoidance of drying and/or transportation costs, and the decrease of waste generation. Moreover, the in situ production of CNF would help the papermakers to determine the relation between the minimum CNF quality and the needs required for a certain recycled paper product, allowing the online control of the properties of the CNF and their adjustment to the production needs. In addition, the negative effects related to the dispersion of the CNF in the pulp suspension would be also avoided. Futhermore, CNF could also be sold in the local market as additive for other industries. In this way, the benefit of this industrial symbiosis will allow papermakers to aford the cost of CNF production.

Therefore, the objective of this study was to evaluate the feasibility of using two different types of recycled pulps—Old Newsprint (ONP) and Old Corrugated Container (OCC)—with 14 wt.% and 11 wt.% ash content, respectively, to simulate the broke streams of paper machines, produce in situ CNF, and study its direct application on the recycled pulp suspension to reinforce the final product, recycled newsprint, and recycled cartonboard, respectively. The production of CNF was studied at different TEMPO (2,2,6,6-tetramethyl-1-piperidinyloxy)-mediated oxidation levels (2.5, 5, 10, and 15 mmol of NaClO per gram of pulp) before the homogenization mechanical process. TEMPO-mediated oxidation is the most common pretreatment to facilitate cellulose defibrillation reducing the energy consumption in homogenization. The obtained CNF were characterized, and several doses of CNF (1, 2, and 3 wt.%) were added to the recycled pulp to evaluate their effect in terms of paper strength enhancement, using a three-component retention and drainage system (TRDS) containing cationic polyamine as coagulant (C), cationic polyacrylamide as flocculant (PAM), and hydrated bentonite (B). The mechanical properties measured on handsheets include tensile strength index, tear strength index, and porosity in the case of addition of CNF from recycled ONP. For CNF from recycled OCC, bursting strength index and short-span compressive strength (SCT) were also measured to evaluate the recycled cartonboard enhancement. Also, the effect of both kinds of CNF on retention and drainage was studied, using different CNF doses and TRDS. 

## 2. Results and Discussion

### 2.1. Characterization of CNF from Recycled ONP and Recycled OCC Pulps

**Figure 2:**
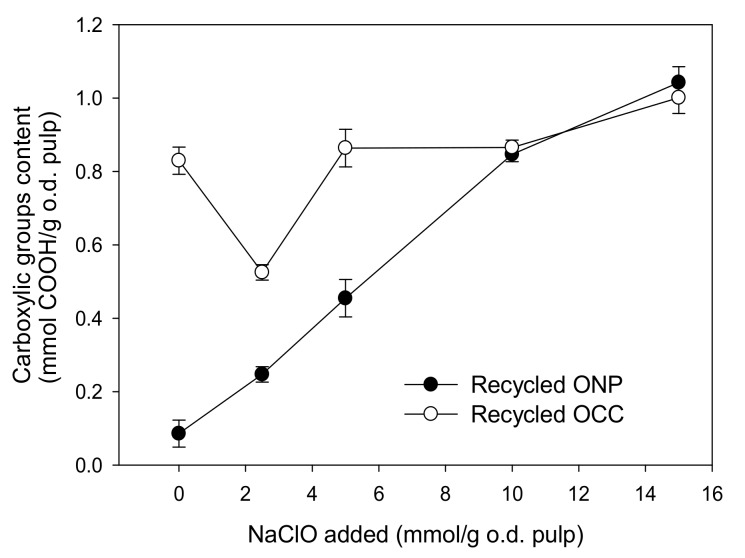
Carboxylic groups in recycled old newsprint (ONP) and old corrugated container (OCC) pulps oxidized by NaClO in presence of 2,2,6,6-tetramethyl-1-piperidinyloxy (TEMPO).

The content of carboxylic groups of the recycled ONP was very low—below 0.2 mmol of COOH/g of dry pulp—compared to the content of carboxylic groups of recycled OCC (~0.85 mmol of COOH/g of dry pulp). In the case of recycled ONP, the COOH level increased linearly with increasing oxidation treatment up to a dose of 15 mmol of NaClO/g of dry pulp (Figure 2). In the case of recycled OCC, the first addition of NaClO decreased the content of carboxylic groups but higher additions produced an increase of the values, obtaining similar content of carboxylic groups to recycled ONP. This was probably due to the higher content of lignin and other impurities present in the recycled OCC (Table 1) that consume part of the NaClO [21].

**Figure 3:**
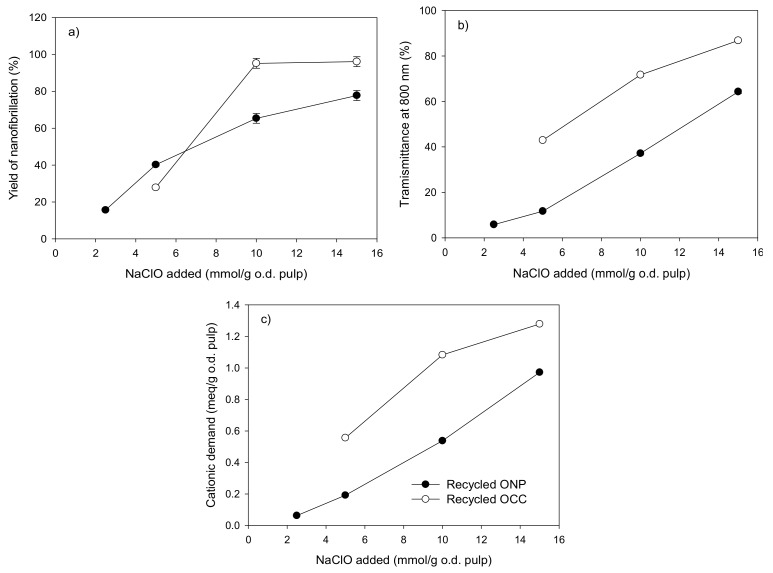
Characterization of CNF from recycled ONP and recycled OCC pulps oxidized by NaClO in the presence of TEMPO. (**a**) Yield of nanofibrillation, (**b**) transmittance at 800 nm, and (**c**) cationic demand.

The yield of nanofibrillation increased with the degree of oxidation, reaching values close to 80% and 100% when 15 mmol of NaClO per gram of dry pulp was used to produce CNF from recycled ONP and recycled OCC, respectively (Figure 3a). As expected, catalytic oxidation with TEMPO and NaClO had an important impact on the transmittance and on the cationic demand of the CNF suspensions, obtaining higher values when the degree of oxidation applied to the recycled pulp increased (Figure 3b,c). The production of CNF from recycled OCC pulps with a degree of oxidation of 2.5 mmol of NaClO per gram of pulp was not possible due to the obstruction of the homogenizer, requiring a minimum degree of oxidation (5 mmol of NaClO per gram of pulp) to carry out the mechanical treatment. 

Results showed that CNF produced from recycled OCC with 10 mmol of NaClO/g of dry pulp or a higher dose of NaClO are more nanofibrillated than CNF produced from recycled ONP, although the chemical pretreatment and homogenization conditions were the same. This effect could be related to the higher amount of lignin of recycled OCC compared to ONP, as shown in the Kappa index values (Table 1). Delgado-Aguilar et al. (2016) [28] and Ferrer et al. (2012) [29] reported that lignin content is a key factor for the nanofibrillation process, obtaining better results when a certain amount of lignin is presented in the pulp. They reported that a good balance between hemicellulose and lignin content facilitates the mechanical defibrillation of the cellulose fibers due to increased swelling caused by hemicelluloses and the formation of mechanoradicals stabilized by residual lignin [28,29,30]. Probably, the amount of hemicelluloses in CNF suspensions from recycled ONP, which has a negligible quantity of lignin, was not enough to reach the higher nanofibrillation yields and transmittances values of the CNF of recycled OCC, even though they had the same carboxylic content at the higher oxidation degrees. In addition, the lower amount of aggregates in the recycled OCC pulp (6.2% less) (Table 1) could also help to improve the defibrillation process.

### 2.2. Effect of CNF Content on Recycled Paper and Cartonboard Properties

**Figure 4:**
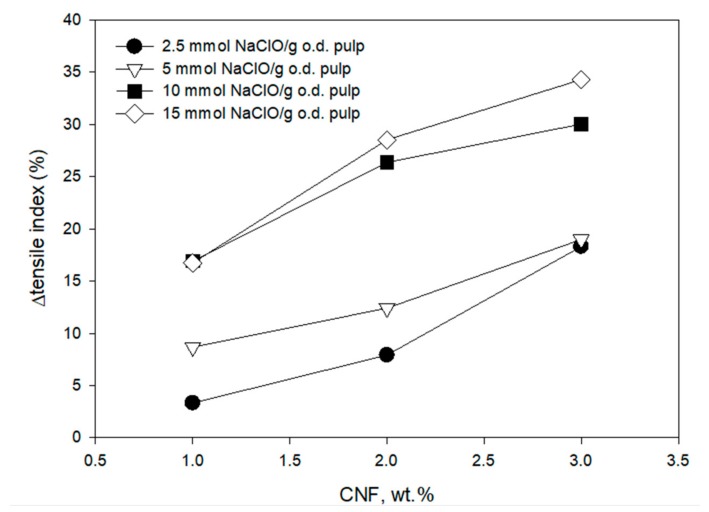
Effect of CNF dose and TEMPO-oxidation degree on tensile index increment of the recycled ONP paper using a three-component retention and drainage system (C-PAM-B).

Once the feasibility of producing CNF from recycled fibers was established, they were applied in mass to evaluate their effectiveness as a reinforcing agent in the recycled pulp (ONP and OCC) using a TRDS for retention of the CNF. The presence of 3 wt.% CNF produced from recycled ONP with low levels of oxidation (2.5 and 5 mmol of NaClO/g of dry pulp) increased the tensile index of the recycled pulp by 18%. CNF with higher degrees of oxidation (10 and 15 mmol NaClO/g of dry pulp) increased the tensile index by 18% and more than 25% using 1 and 2 wt.% of CNF, respectively (Figure 4). A higher increase in the tensile strength (28.5% and 34.5% with 2 and 3 wt.% CNF, respectively) was obtained with the addition of CNF produced with the higher oxidation degree (15 mmol of NaClO/g of pulp), these being the highest nanofibrillated fibers in terms of nanofibrillation yield and transmittance. The CNF obtained from the recycled ONP pulp, oxidized with 10 mmol of NaClO/g of pulp, appears to be adequate for the improvement of mechanical properties of recycled paper, as there is high tensile index enhancement, similar to that obtained with 15 mmol of NaClO/g of pulp (only 5% less), but requiring 33% less NaClO. Results showed that there is a relationship between the quality of the CNF and the improvement in the mechanical properties, but this relationship is complex and it is not proportional. In other studies the quality of the CNF did not have a simple direct relation on the mechanical properties of the recycled paper [21] due to the influence of other factors, such as the dispersion of the CNF in the pulp suspension, the interaction between the CNF and the rest of the components present in the pulp, as well as the flocculation processes that may occur and affect the uniformity of the final paper [31]. This shows the importance of the application methodology for the CNF industrial application.

**Figure 5:**
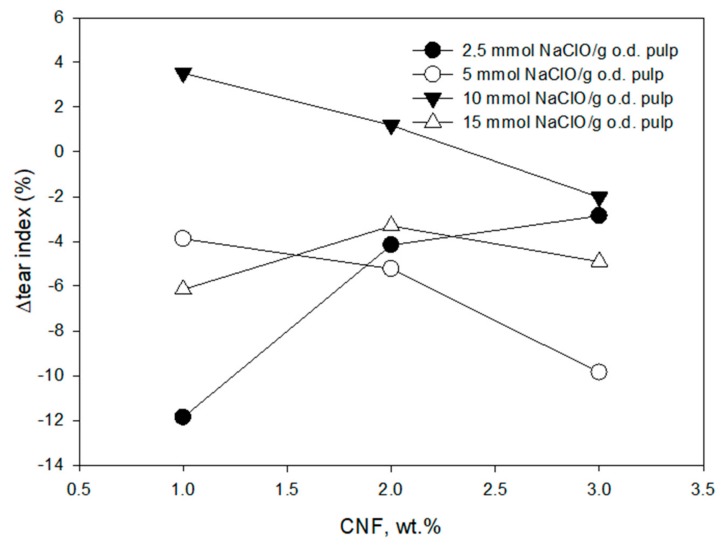
Effect of CNF dose and TEMPO-oxidation degree on tear index increment of the recycled ONP paper using a three-component retention and drainage system (C-PAM-B).

**Figure 6:**
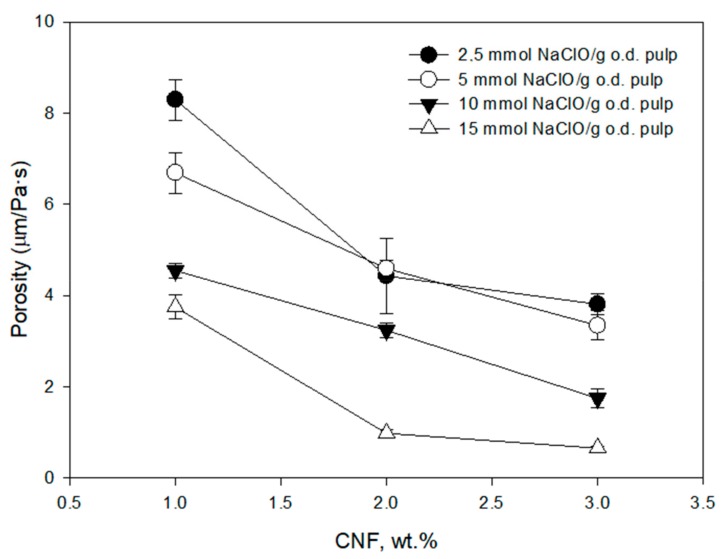
Effect of CNF dose and TEMPO-oxidation degree on porosity of the recycled ONP paper using a three-component retention and drainage system (C-PAM-B).

The effect on the tear index is not very significant although there were improvements achieved at 10 mmol of NaClO/g of pulp for 1 and 2 wt.% of CNF (Figure 5). As expected, the porosity of the handsheets is lower when the amount of CNF increases and the lowest values of porosity were obtained at high TEMPO-mediated oxidation level due to the high yield of the nanofibrillation achieved at these conditions (Figure 6). Both an increase in CNF content and a higher fibrillation produces a higher block of pores that reduces the porosity. The reduction of porosity produce a higher interaction in the hydrogen bounding improving the tensile strength. However, the tear strength does not show a clear trend due to the dependence of several factors. On the one hand, the tear index improves with the hydrogen bounding that increases with CNF content and the block of pores. However, the same property is reduced with short fibers that facilitate the tear of the sheet. 

Comparing the improvement in tensile strength with published data, available only for CNF produced from virgin and agriculture residues pulps, the conclusion is that the effect depends on both the type of CNF and the used retention system. Merayo et al. (2017) [32] used bleached Eucalyptus pulp to produce CNF (TEMPO-5 mmol of NaClO/g of pulp and six steps of homogenization at 600 bars) and the tensile index of newsprint increased around 60%, using 3 wt.% of CNF and the same retention system as in this study. In a similar study, Delgado-Aguilar et al. (2015) [16] obtained an increase up to 52% when cationic starch was used as retention system; although the real improvement by the 3% CNF addition was lower since cationic starch is also a strength agent. When corn stalk pulp was used to produce CNF (TEMPO-15 mmol of NaClO/g of pulp and six steps of homogenization at 600 bars) tensile index of newsprint also increases by 60% using the same TRDS as in this paper [32]. On the other hand, when 3 wt.% of CNF (obtained by bleaching with 8 wt.% NaClO/g of pulp and a homogenization cycle of three steps at 300 bars, three steps at 600 bars, and three steps at 900 bars) from sawdust of pine; Eucalyptus and triticale were used with the same TRDS and the tensile index of newsprint increased by 15%, 8%, and 3.5%, respectively [33]. Even more interesting is the comparison of the obtained data with the use of CNC, produced and applied to the same recycled newspaper. In this case, similar increments up to 30% in the tensile strength index were achieved when 3 wt.% of CNC was added into the recycled pulp using a similar polyacrylamide-based retention system [24]. 

**Figure 7:**
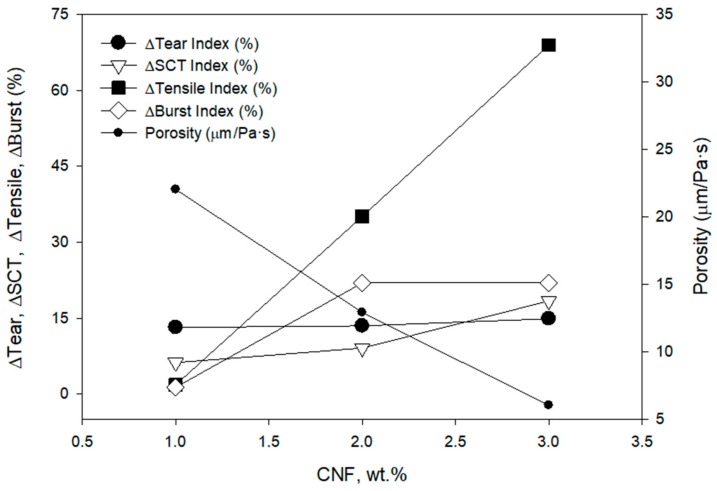
Effect of CNF dose on mechanical properties of the recycled OCC paper using CNF produced from recycled OCC using 10 mmol of NaClO per gram of pulp before the homogenization and a three-component retention and drainage system (C-PAM-B).

CNF from recycled OCC were prepared by TEMPO-mediated oxidation using 10 mmol of NaClO per gram of pulp before the homogenization because those were the optimal mechanical properties of the handsheets obtained previously for recycled ONP. The effect of the CNF on the short-span compressive test (SCT) and burst index of the cartonboard—the same raw material as the one used to prepare CNF, besides tensile index, tear index, and porosity—was studied at 1, 2, and 3 wt.% doses of CNF. The retention and drainage system C-PAM-B was added to the pulps, and handsheets were formed and characterized. In this case, 3 wt.% CNF increased the tensile index above 60%, and decreased the porosity, as it was expected (Figure 7). Burst index and SCT increased above 15% for a 3 wt.% CNF dose. The mechanical properties obtained are similar than the results of Balea et al. (2016) [14] that used virgin pulps, namely bleached Eucalyptus and pine, to produce CNF using cationic starch as retention system. They applied 3 wt.% CNF reaching only 20–25% tensile index improvement; however, they achieved 30% and 37% increases in burst and tensile indexes, respectively.

### 2.3. Effect of CNF on Retention and Drainage Process

**Figure 8:**
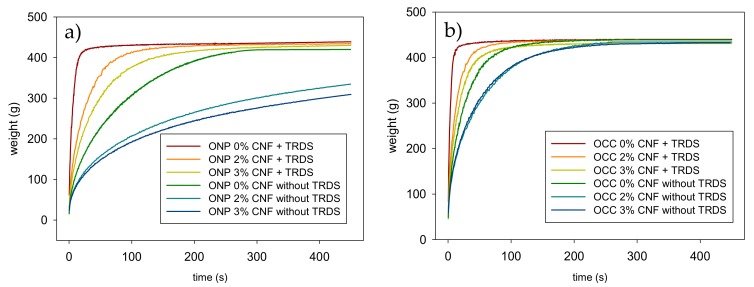
Effect of CNF dose and three-component retention and drainage system on the drainage process. (**a**) ONP and (**b**) OCC.

**Figure 9:**
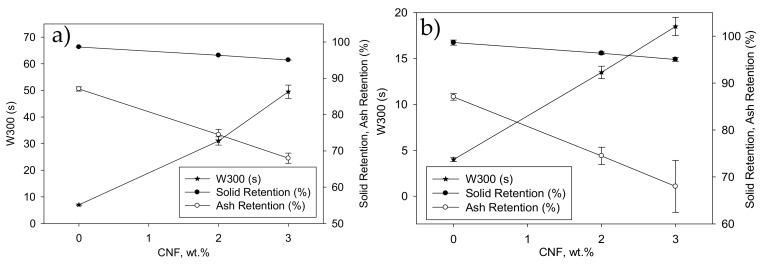
Effect of drainage time (W300), solid retention, and ash retention of the handsheets with different doses of CNF and TRDS. (**a**) Recycled ONP pulp and (**b**) recycled OCC pulp.

The effect of the addition of CNF was assessed on the retention and drainage process of recycled ONP and recycled OCC with and without a retention system. In both cases, drainage effect was studied only with CNF oxidized with 10 mmol of NaClO/g of pulp before the homogenization. Figure 8 shows the drainage curves of experiments performed with and without TRDS for recycled ONP (Figure 8a) and recycled OCC (Figure 8b). To compare drainage results, drainage time was calculated when 300 g of water were drained (W300). Figure 9 shows the effect of CNF dose on the W300 time, the total solid retention and ash retention in recycled ONP and OCC pulps (Figure 9a,b, respectively) using TRDS. 

In general, the drainage time of the recycled ONP pulp was lower than OCC in all conditions studied, probably due to the higher amount of fines that the recycled ONP pulp has compared to the recycled OCC pulp (Table 1). Cellulose fines usually consist of a very complex and heterogeneous set of materials, thus a certain fraction of fines is similar to cellulose microfibers [19]. The drainage results obtained in this study are according to Taipale et al. (2010) [19] and Johnson et al. (2016) [34], which demonstrated that lower content of fines in the pulp decreased the dependence of the drainage time with the CNF content. In both recycled pulps, higher doses of CNF absorbed more water in the pulp, making more difficult the drainage process, thus increasing the W300 by 334% and 77% when 3 wt.% CNF from recycled ONP and recycled OCC were added into the pulp in absence of TRDS. However, this effect can be reduced by 48% and 30%, respectively with the incorporation of TRDS as retention agent. These results are according to several authors, which demonstrated that the addition of CNF into a pulp suspension gets worse the drainage rate but this effect can be counteracted by the addition of different retention systems [19,21,32,34]. 

For total solids retention, determined through gravimetric analysis, the addition of both CNF reduced their retention, reaching a minimum of 95% when a 3 wt.% of CNF was added to the recycled pulps. Finally, the trend of the ash retention was similar as that of total solids retention, decreasing as CNF content increased. Both recycled ONP and OCC pulps without CNF have an ash retention ~85%, whereas these values decrease until 70 wt.% when a 3 wt.% of CNF was added (Figure 9).

## 3. Materials and Methods

### 3.1. Materials

Recycled old newsprint (ONP) and old corrugated containers (OCC), manufactured by Holmen Paper Madrid (Madrid, Spain) and Räpina Paperivabrik AS (Räpina, Estonian), respectively, were used as raw materials to simulate broke streams to produce both the CNF and the recycled pulps in which CNF were added at different dosages. Table 1 presents the results of the morphological analysis of the ONP and OCC pulps, obtained using a Morfi analyzer V7.9.13E (Techpap, France).

A three-component retention and drainage system (C-PAM-B) was assessed as it is commonly used in the recycled paper industry. The doses used for the laboratory experiments are based on industrial recommendations [35]. The three-component system selected was a bentonite-based microparticle system that contains: 1.25 mg/g of cationic polyamine as coagulant (cationic charge density of 0.035 meq/g and high molecular weight); 0.75 mg/g of cationic polyacrylamide (PAM) with high molecular weight (cationic charge density of 3.66 meq/g) as flocculant; and 1.7 mg/g of hydrated bentonite clay, all of them supplied by BASF (Ludwigshafen, Germany).

### 3.2. Methods

#### 3.2.1. CNF Production and Characterization

CNF produced from recycled ONP and OCC were obtained by TEMPO -mediated oxidation by using 2.5, 5, 10, and 15 mmol of NaClO/g of pulp. The reaction was performed at room temperature, maintaing pH at ~10, and using a NaOH solution at 0.5 M [36]. After the oxidation process, the pulp was cleaned through filtration steps using tap water to reach a neutral pH. Finally, CNF were homogenized in a PANDA PLUS 2000 laboratory homogenizer (GEA Niro Soavi, Italy) at 600 bar. The number of passes through the homogenizer was the required to obtain a gel suspension of CNF and depend on the oxidation grade (in the case of CNF with 2.5 and 5 mmol of NaClO, 15 passes were applied, for 10 mmol of NaClO the number of passes was 5 and, finally, 3 passes were applied for CNF with 15 mmol of NaClO). 

To characterize the oxidized cellulose pulp the amount of carboxyl groups was measured as an indicator of the oxidation degree achieved after TEMPO-mediated oxidation by conductimetric titration according to Balea et al (2016) [15] and calculated based on the method development by Habibi et al. (2006) [37]. As for CNF characterization, nanofibrillation yield was measured in a diluted CNF suspension (0.1 wt.%) by centrifugation at 4500× *g* for 30 min. The nanofibrillated fraction is isolated in the supernatant from the nonfibrillated fraction deposited in the sediment. Transmittance of the CNF suspensions diluted at the same concentration as previously were measured between 400 and 800 nm of wavelength using a Cary 50Conc UV–visible spectrophotometer (Varian Australia Pty Ltd, Victoria, Australia). Cationic demand was measured by colloidal titration of the diluted suspension at 0.05 wt.%, with 0.001 N polyDADMAC, using a Mütek PCD04 particle charge detector (BTG Instruments GmbH, Herrsching, Germany). Finally, polymerization degree was calculated from the limiting viscosity number of CNF suspensions, using cupriethylendiamine as a solvent and determined by the international standard ISO5351/1, based on Mark–Houwink–Sakurada (MHS) equation and the studies of Marx-Figini (1978) [38] and Henriksson et al. (2008) [39].

#### 3.2.2. Handsheet Preparation and Characterization

Recycled pulps (ONP and OCC) were prepared through disintegration of 20 g of dry recovered paper in 2000 mL of water using a Messmer pulp disintegrator (Mavis Engineering Ltd, London, UK). The recovered paper with the correspondent amount of CNF (1.0, 2.0, and 3.0 wt.%) was left to soak at least 24 h before disintegration to favor swelling. A three-component retention system was added to the pulp (1.25 mg/g of coagulant, 0.75 mg/g cationic polyacrylamide as flocculant, and 1.7 mg/g hydrated bentonite clay based on industrial recommendations). The pulp was used to prepare handsheets with basis weight of 80 g/m^2^ for both recycled papers in a normalized handsheet former Rapid-Köthen (ISO 5269/2, DIN 54 358). 

Mechanical properties were determinated by measuring tensile strength index (kN·m/kg), tear strength (mN), porosity (μm/Pa·s), short-span compressive test (SCT) index (N·m/g), and bursting strength index (kPa·m^2^/g). Tensile strength was measured in a MTS Criterion Mode 43 from MTS Systems Corporation (Eden Prairie, MN, USA), following ISO 1924-3 (2014) standard. Tear strength was determined according to ISO 1974:2012 using a tearing resistance tester. Bendtsen porosity (μm/Pa·s) was measured with a Bendtsen Porosity Tester nº 8699 from Andersson & Sørensen (Copenhague, Denmark) according to ISO 5636-3 (2013). To measure the cross directional short-span compressive strength a short span compression tester (Messmer Büchel, Veenendaal, The Netherlands) was used according to TAPPI T826 standard (2013). Finally, bursting strength was measured in a Messmer Büchel digital hydraulic board burst tester according to standard ISO 2759 (Veenendaal, The Netherlands). 

#### 3.2.3. Retention and Drainage Measurements

Drainage measurements of the pulp suspensions were carried out in a MütekTM DFR-05 (DFR) from BTG Instruments (Säffle, Sweden), which provided the drainage curves of the pulp when it is drained by gravity through 150 mesh. Experiments were performed with 500 mL of pulp suspension at 0.5 wt.% consistency. First, the pulp suspension was placed in an agitation chamber and it was agitated at 300 rpm. After 30 s of initial stirring, the retention aids were added to the pulp in the DFR (coagulant was firstly added, then, at consecutive intervals of 30 s, cPAM and bentonite were also added). Finally, after a further 30 s of mixing, the stirring was stopped, and the filtration step began monitoring and recording the weight of the drained on real time. Solids retention was measured by gravimetric analysis of the total solids in the drained water at 105 °C, and ash retention was determined by incineration at 525 °C (ISO 1762, 2015). 

## 4. Conclusions

In situ production of CNF, from recycled ONP and OCC that simulate broke streams of the paper machines, is feasible in terms of improving the final product quality. The potential increase of the strength properties depends on the CNF properties, which are linked to the CNF production, the CNF dosage, and the retention system used. Implementation of this strategy would reduce the costs and difficulties of CNF transportation and application, valorizing the waste streams containing cellulose and contributing to the sustainability and circular economy in the process. Furthermore CNF could be also sold in the local market for other applications to contribute to the economy of the process. 

The use of 10 mmol of NaClO/g of pulp in TEMPO-mediated oxidation before the homogeneization is enough to improve mechanical properties of recycled ONP pulp with an increase of tensile index around 30% with a 3 wt.% of CNF and with slightly impact on tear index. These results are very similar to the application of 3 wt% CNC obtained from the same raw material and applied to the same newsprint pulp. A further increase in the oxidation conditions to produce CNF do not improve efficiently the mechanical properties.

On the other hand, the optimal conditions to prepare CNF from ONP were also applied to prepare CNF from OCC. In this case, tensile index increased above 60% with a 3 wt.% CNF, whereas tear, SCT, and bursting indexes raised ~15–20%. Finally, CNF from both cellulose sources had worse drainage, but this effect was effectively counteracted with the optimization of the three-component retention system used.

## Figures and Tables

**Table 1:** Morphology of recycled old newspaper.

Item	Units	Recycled ONP	Recycled OCC
Fibers			
Length weighted in length	(μm)	861	1054
Average width	(μm)	21.4	22.2
Coarseness	(mg/m)	0.141	0.159
Microfibrils	(%)	1.72	1.35
Broken ends	(%)	37.2	34.1
Average angle	(º)	130.3	133.5
Kinked fibers	(%)	13.70	13.54
Average curl	(%)	5.89	5.64
**Pulps**			
Kappa index		40	72
Fibers	(number × 10^6^/g)	15.82	11.80
Aggregates	(number/g)	98,837	92,667
Fines	(number/g)	118,646	92,322
